# Tissue‐specific differences in HIV DNA levels and mechanisms that govern HIV transcription in blood, gut, genital tract and liver in ART‐treated women

**DOI:** 10.1002/jia2.25738

**Published:** 2021-07-08

**Authors:** Sara Moron‐Lopez, Guorui Xie, Peggy Kim, David A Siegel, Sulggi Lee, Joseph K Wong, Jennifer C Price, Najwa Elnachef, Ruth M Greenblatt, Phyllis C Tien, Nadia R Roan, Steven A Yukl

**Affiliations:** ^1^ Department of Medicine University of California San Francisco (UCSF) San Francisco CA USA; ^2^ Department of Medicine San Francisco VA Medical Center San Francisco CA USA; ^3^ Department of Urology University of California San Francisco (UCSF) San Francisco CA USA; ^4^ Gladstone Institutes San Francisco CA USA

**Keywords:** HIV, virus latency, DNA, RNA, women, intestines, cervix uteri, liver

## Abstract

**Introduction:**

Sex‐specific differences affect multiple aspects of HIV infection, yet few studies have quantified HIV levels in tissues from women. Since an HIV functional cure will likely require a major reduction of infected cells from most tissues, we measured total and intact HIV DNA and the HIV transcription profile in blood, gut, genital tract and liver from HIV‐positive antiretroviral therapy (ART) ‐treated women.

**Methods:**

Peripheral blood mononuclear cells (PBMC) and biopsies from the gastrointestinal (ileum, colon, rectosigmoid +/‐ liver) and genital (ectocervix, endocervix and endometrium) tracts were collected from 6 ART‐treated (HIV RNA < 200 copies/mL) women. HIV DNA (total and intact) and levels of read‐through, initiated (total), 5’elongated, polyadenylated and multiply spliced HIV transcripts were measured by droplet digital PCR. Immunophenotyping of cells was performed using Cytometry by time of flight (CyTOF).

**Results:**

We detected total HIV DNA in all tissues and intact HIV DNA in blood, ileum, colon, rectosigmoid and ectocervix. Initiated HIV transcripts per provirus were higher in PBMC and endometrium than in ileum, colon, rectosigmoid, ectocervix or endocervix, and higher in the rectum than either ileum or colon. 5’Elongated HIV transcripts per provirus were comparable in PBMC and endometrium, but higher than in gut or cervical samples. Polyadenylated and multiply spliced HIV transcripts were detected in PBMC (6/6 and 3/6 individuals respectively), but rarely in the tissues.

**Conclusions:**

These results suggest tissue‐specific differences in the mechanisms that govern HIV expression, with lower HIV transcription in most tissues than blood. Therapies aimed at disrupting latency, such as latency‐reversing or latency‐silencing agents, will be required to penetrate into multiple tissues and target different blocks to HIV transcription.

## 1 INTRODUCTION

Globally, in 2019 there were approximately 38 million adults and children living with human immunodeficiency virus (HIV), of whom 51% were women aged 15 and over [1]. Sex‐specific differences manifest in immune‐mediated protection from pathogens, vaccine efficacy and prevalence of most autoimmune diseases [2, 3]. Sex‐immunologic dimorphisms are driven by multiple biological factors, including genetic differences derived from the chromosomal complement [4, 5], sex‐specific epigenetics [6, 7] and sex hormones [8, 9]. Biological sex also affects various aspects of HIV infection and progression [10, 11], such as plasma viral load [12, 13, 14] and immune activation. For example, levels of plasma HIV RNA proximal to seroconversion are higher in men than women [15, 16, 17, 18, 19, 20, 21], although these differences attenuate with disease progression. In contrast, women exhibit higher T‐cell activation [22], interferon‐α production after toll‐like receptor 7 stimulation [22, 23] and expression of interferon‐stimulated genes when matched by level of plasma viraemia [24].

One of the main obstacles to curing HIV is the viral reservoir, which includes latently infected cells that do not produce virus constitutively but can generate infectious virus upon activation [25, 26, 27]. Antiretroviral therapy (ART) is unable to eliminate the latent HIV reservoir, which persists for many years [28, 29, 30, 31]. Although cells from the peripheral blood have provided many insights into HIV persistence, 98% of total‐body CD4^+^ T cells reside in lymphoid tissues [32, 33, 34]. Most tissues harbour CD4^+^ T cells, although the contribution of each tissue to the whole‐body HIV reservoir size varies due to tissue‐specific differences in the numbers, phenotypes and infection frequencies of CD4^+^ T cells and other infectable cells [32, 35, 36, 37]. The gut harbours a high proportion of HIV‐infected cells in ART‐treated men [35, 38, 39, 40], and HIV has been detected in the liver of ART‐treated individuals [41, 42]. Furthermore, HIV DNA and RNA have been detected in the genital tract and genital secretions from ART‐treated men and women [32, 43, 44, 45, 46, 47, 48, 49, 50, 51, 52, 53, 54, 55, 56]. However, few studies have quantified levels of HIV persistence and expression in the female gastrointestinal tract [57] and none in more than one tissue of the female genital tract. Here, we measured the total and intact HIV DNA, HIV transcription profile and immunophenotypes of CD4^+^ T cells in the blood, gut, liver and genital tract from ART‐treated women.

## 2 METHODS

### 2.1 Study participants

Participants were enrolled from the Northern California site of the Women’s Interagency HIV Study (WIHS), a United States (U.S.) multicentre prospective cohort [58]. Among the sixteen ART‐treated cisgender premenopausal women eligible for this substudy, six agreed to participate. Peripheral blood, gut biopsies (ileum, colon, rectosigmoid) and genital tract samples (biopsies from endometrium and ectocervix, plus endocervical curettage) were collected from all six participants during the midluteal phase of the ovulatory cycle, as determined by urine luteinizing hormone detection and verified by blood progesterone. Blood, gut and genital tissues were obtained at the same study visit, between May 2017 and October 2019.

Liver biopsies were also collected from three out of the six participants (Figure S1) within a year prior to the other samples as part of another substudy evaluating non‐alcoholic fatty liver disease in people living with HIV. Liver biopsies were offered to women without hepatitis B or C virus infection if there was evidence of possible fatty liver (Magnetic resonance spectroscopy‐measured liver fat fraction ≥0.05, Controlled Attenuation Parameter ≥238 dB/m, elevated liver enzymes on at least two occasions without evidence of other underlying liver disease, or liver stiffness ≥7.1 kPa). Both studies were approved by the local University of California, San Francisco Institutional Review Board. Each participant provided written informed consent.

### 2.2 Blood/tissue processing

Cytometry by time of flight (CyTOF) analysis was conducted on a subset of the samples in order to calculate the frequencies of CD4^+^ T cells in the specimens, and to assess the extent of blood contamination in tissue specimens. Peripheral blood mononuclear cells (PBMC) were isolated through Ficoll–Paque density gradient sedimentation. 30 mL FACS buffer (PBS with 2%FBS and 2 mM EDTA) was added to the blood, followed by slow addition of 10 mL Ficoll (Stemcell Technologies, Cambridge, MA, USA) to the bottom of the tube. Cells were then centrifuged at 2,000 rpm at room temperature without braking using an Allegra X‐12 (Beckman Coulter, Brea, California, USA). PBMCs were transferred to a new tube, washed three times with FACS buffer, and treated for 10 minutes at room temperature with Red Lysis Buffer (BioLegend, San Diego, CA, USA). The cells were then washed twice with RPMI 1640 supplemented with 10% FBS and 1% Penicillin‐Streptomycin‐Glutamine and prepared for CyTOF.

Tissue specimens were processed into single‐cell suspensions as described previously [59]. Each tissue was incubated with 10 mL intra‐epithelia lymphocyte (IEL) digestion buffer (Ca^2+^‐free and Mg^2+^‐free PBS with 10 mM DTT (Sigma‐Aldrich, St. Louis, MO, USA), 5 mM EDTA (Thermo Fisher Scientific, Waltham, MA, USA), 10 mM HEPES (Thermofisher) and 5% FBS) for 20 minutes at 37°C under constant rotation. Cells in the supernatant (IELs) were then passed through a 70 μm strainer (Fisher), washed with Rinse Buffer (RB) (RPMI 1640 with 10 mM HEPES and 5% FBS), resuspended in media and stored at 37°C. Tissues were then treated a second time with 10 mL digestion buffer for 20 minutes at 37°C under constant rotation, passed through another 70 μm strainer, washed with RB and combined with the IELs. The remaining tissues (consisting primarily of lamina propria) were then transferred to MACS C tubes (Miltenyi Biotec) in digestion solution (6 mL RPMI‐1640, 10 mM HEPES, 5% FBS, 6 mg collagenase (Worthington‐Biochemical Corp, Lakewood, NJ, USA) and 7.5 μg/mL DNAse (Sigma‐Aldrich)), incubated for 30 minutes at 37°C under constant rotation, and vortexed for 10 seconds. These lamina propria cells were then dissociated using the gentleMACS^TM^ Dissociator (Miltenyi, program: m_spleen), followed by mechanical dissociation by 10 passes through a blunt 20G needle (Becton Dickinson). The cells were then filtered through a 70 μm strainer, washed with RB and passed through a new 70 μm strainer, washed and filtered again. Finally, the cells were treated with 1 mL DNase solution (6 mL RPMI 1640, 10 mM HEPES, 5% FBS and 7.5 μg/mL DNAse (Sigma‐Aldrich)) for 30 minutes at 4°C, washed once with RB, and combined with the IELs.

For quantification of HIV DNA and HIV transcripts, nucleic acids were extracted from PBMCs and flash‐frozen biopsies using TRI Reagent^®^ (Molecular Research Center Inc., Cincinnati, OH, USA) [60].

### 2.3 CyTOF preparation

CyTOF was conducted similar to methods previously described [61, 62]. PBMCs and single‐cell suspensions of tissues were treated for 1 minutes with 25 μM cisplatin (Sigma) [live/dead discriminator] diluted in PBS, quenched with CyFACs (PBS with 0.1% BSA and 0.1% sodium azide) and then fixed with 2% paraformaldehyde (PFA) for 10 minutes at room temperature. Cells were washed twice with CyFACs and then frozen at −80°C until staining. For staining, cells were first barcoded using the Cell‐ID^TM^ 20‐Plex PD Barcoding kit (Fluidigm, South San Francisco, CA, USA) and then blocked for 15 minutes at 4°C with 1.5% mouse sera (Thermo Fisher Scientific), 1.5% rat sera (Thermal Fisher) and 0.3% human AB sera (Sigma‐Aldrich). Next, the cells were washed twice with CyFACs, stained for 45 minutes at 4°C with antibodies targeting surface antigens (Table S1), washed and then fixed overnight at 4°C in 2% PFA diluted in PBS. Cells were then permeabilized for 30 minutes at 4°C with Foxp3 Fix/Permeabilization Buffer (Fisher Scientific), washed twice with Permeabilization Buffer (Fisher Scientific, Waltham, MA, USA) and blocked for 15 minutes at 4°C with 15 μL mouse Serum and 15 μL rat serum diluted into 80 μL Permeabilization Buffer. Next, cells were washed with Permeabilization Buffer, stained for 45 minutes at 4°C with antibodies targeting intracellular antigens (Table S1) and incubated for 20 minutes at room temperature with a 1:500 dilution of DNA intercalator solution (Fluidigm). Finally, cells were washed with CyFACS, washed with Cell Acquisition Solution (CAS, Fluidigm), resuspended in 1x EQ^TM^ Four Element Calibration Beads (Fluidigm) diluted in CAS and analysed on a CyTOF 2 (Fluidigm). Paired PBMCs and tissues were stained and run at the same time.

### 2.4 Total and intact HIV DNA

**Figure 1:**
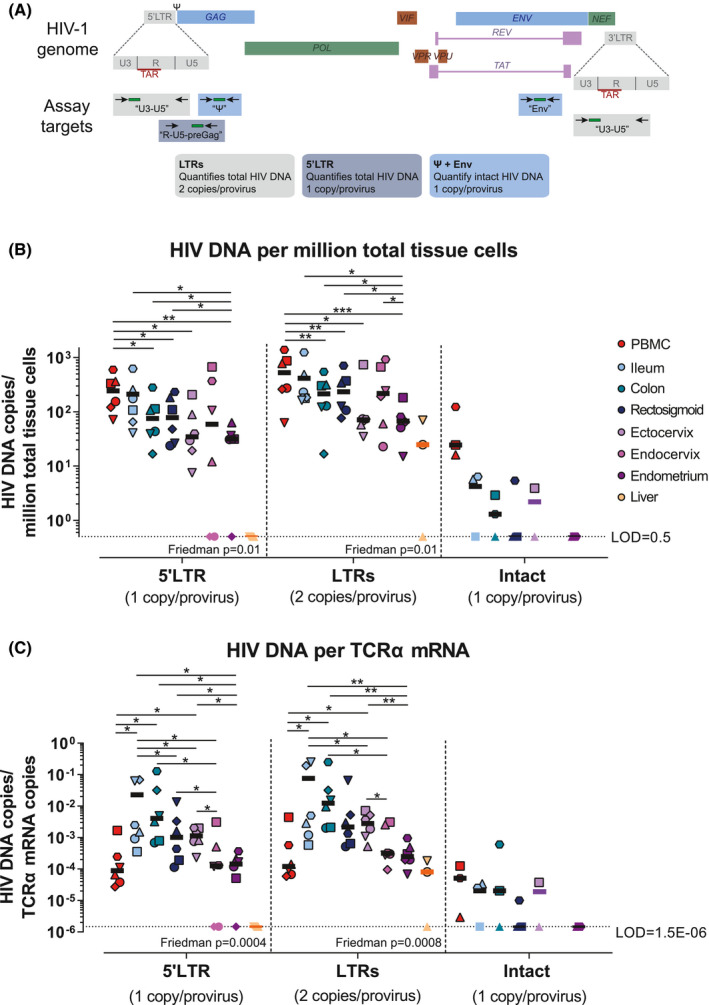
Total and Intact HIV DNA. **(A)** Schematic representation of the HIV genome and the assays used to quantify total and intact HIV DNA. **(B)** Quantification of total (LTRs and 5’LTR) and intact (Ψ+Env) HIV DNA per million total tissue cells and **(C)** per TCRα mRNA expression (normalization by T‐cell content). Symbol colours denote different tissues; symbols/shapes show different participants; symbols without borders indicate values below the limit of detection; FDR‐corrected *p*‐values from multilevel mixed‐effects negative binomial regression are represented as *p ≤ 0.05, **p ≤ 0.001 and ***p ≤ 0.0001. Friedman *p* is the Friedman test *p*‐value.PBMC, Peripheral blood mononuclear cells.

Total HIV DNA levels (LTRs [U3‐U5] and 5’LTR [R‐U5‐preGag]) were quantified in duplicate using droplet digital PCR (ddPCR) (Bio‐Rad) as described [63], normalizing to million cells by quantification of the cellular gene TERT. Intact HIV DNA (Packaging signal [Ψ] and Env [RRE]) was quantified in eight replicates from three out of the six participants (the ones with highest total HIV DNA) using ddPCR as described [64], including the use of the hypermutated probe for Env region (Figure 1A). DNA samples from HIV‐negative donors were used to assess for false positives.

### 2.5 Level of HIV transcription

**Figure 2:**
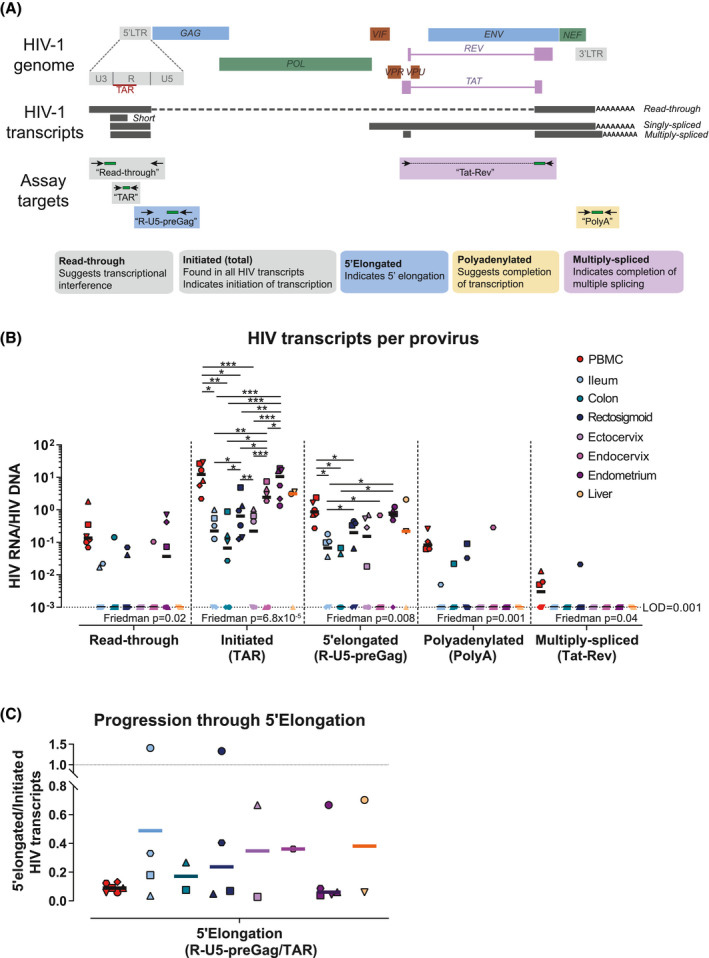
Levels of HIV transcription. **(A)** Schematic representation of the HIV genome, the HIV transcripts and the assays used to quantify HIV RNA. **(B)** Levels of each HIV transcript per provirus (HIV RNA/DNA). **(C)** Progression through HIV 5’elongation (5’elongated/initiated HIV RNA). Symbol colours denote different tissues; symbols/shapes show different participants; symbols without borders indicate values below the limit of detection; FDR‐corrected *p*‐values from multilevel mixed‐effects negative binomial regression are represented as *p ≤ 0.05, **p ≤ 0.001 and ***p ≤ 0.0001. Friedman *p* is the Friedman test *p*‐value.PBMC, Peripheral blood mononuclear cells.

Cell‐associated HIV transcripts (read‐through, initiated [TAR], 5’elongated [R‐U5‐preGag], polyadenylated [polyA] and multiply spliced [Tat‐Rev]) were quantified in duplicate using reverse transcription ddPCR (Bio‐Rad) as previously described [63] and normalized to million cells using the quantification of the cellular gene TERT (Figure 2A). Progression through HIV 5’elongation was determined by the ratio of 5’elongated to initiated transcripts. RNA samples from HIV‐negative donors were run in parallel to assess for false positives.

### 2.6 Statistics and data analysis

Wells without positive droplets were quantified assuming the detection limit of 1 copy/well. Friedman tests were performed using R to determine that there is significant variation in HIV levels across tissues. Post hoc tests for individual tissue comparisons were performed using multilevel mixed‐effects negative binomial regression using Stata, and corrected for multiple comparisons by Benjamini–Hochberg and Bonferroni methods using R. The number of copies of a given HIV transcript (or HIV DNA) was modelled as the outcome variable, tissue type was a covariate and a random offset was assumed for each individual to ensure that the analysis proceeds in a pairwise manner. The exposure variable was defined as the number of tissue cells for Figure 1B, the number of copies of T‐cell receptor α (TCRα) for Figure 1C, or the number of HIV DNA copies for Figure 2B. Data were also analysed in pairwise fashion using the Wilcoxon signed‐rank test, using GraphPad Prism version 7.0, and results were similar to the multilevel mixed‐effects negative binomial regression analysis (Figure S2). The liver was excluded from the above analyses due to the small sample size. CyTOF data export was conducted using FlowJo (BD Biosciences) and Cytobank software. tSNE analyses were performed using the Cytobank software with default settings. All cellular markers not used in the upstream gating strategy were included in generating the tSNE plots, while non‐cellular markers (live/dead stain) were excluded.

### 2.7 Role of the funding source

The study sponsors had no role in the study design, in the collection, analysis and interpretation of data, in the writing of the report or in the decision to publish.

## 3 RESULTS

### 3.1 Characteristics of the study participants

The study participants included six women who had been living with HIV for a median [range] of 15.2 [7.4 to 23.5] years and taking ART for 11.9 [7.3 to 23.0] years (Table 1). Three participants were on a protease inhibitor‐based ART and the other three on an integrase inhibitor‐based ART. The median plasma viral load was 35 [20 to 185] HIV‐1 RNA copies/mL, the median CD4 count was 938 [595 to 1092] cells/mm^3^ and the median CD4/CD8 ratio was 1.0 [0.5 to 2.4].

**Table 1:** Characteristics of study participants

	HIV‐positive ART‐treated cisgender women (n = 6)
Age (years, median [range])	47.3 [40.8 to 52.3]
Time since HIV diagnosis (years, median [range])	15.2 [7.4 to 23.5]
Time on ART (years, median [range])	11.9 [7.3 to 23.0]
PI+2NRTI‐based ART (n [%])	3 [50]
InI+2NRTI‐based ART (n [%])	3 [50]
Viral load (HIV‐1 RNA cp/mL plasma, median [range])	35 [20 to 185]
Nadir CD4 (cell/mm^3^, median [range])	305 [216 to 356]
Absolute CD4 count (cell/mm^3^, median [range])	938 [595 to 1,092]
Percentage CD4 (%, median [range])	41 [26 to 55]
CD4/CD8 ratio (median [range])	1.0 [0.5 to 2.4]

Abbreviations: ART, antiretroviral therapy; NRTI, Nucleoside reverse transcriptase inhibitor

### 3.2 The gut, genital tract and liver are all sites of HIV persistence in ART‐treated women

To measure HIV persistence, we quantified total HIV DNA (U3‐U5 region [found in both LTRs] or R‐U5‐preGag region [specific for 5’LTR]) in PBMC, gut (ileum, colon, rectosigmoid), genital tract (ectocervix, endocervix, endometrium) and liver. In the women in whom we detected the highest levels of total HIV DNA, we also quantified intact HIV DNA (via the Intact Proviral DNA Assay [IPDA]) in PBMC, ileum, colon, rectosigmoid, ectocervix and endometrium (Figure 1A). Using assays for the 5’LTR (1 copy/provirus) and both LTRs (2 copies/provirus), total HIV DNA was detected in all tissues and normalized to copies/million total cells (Figure 1B). Total HIV DNA per million cells was higher in PBMC than in the colon, rectum, ectocervix or endometrium, and was higher in all gut sites compared to endometrium. While HIV DNA did not differ significantly between gut tissues, it was higher in endocervix than in endometrium (False Discovery Rate [FDR] corrected *p* < 0.05; Figures 1B and Figure S4A). These results suggest that the gut, genital tract and liver are all sites of HIV persistence in ART‐treated women and that the total tissue burden differs among tissues.

Intact proviruses were detected in PBMC from three out of three women, in ileum and colon from two of three women and in rectosigmoid and ectocervix from one of three women (Figure 1B). The number of analysed cells was similar among all tissues except endocervix (Figure S3A). These data suggest that HIV‐infected cells in both the gut and female genital tract harbour intact proviruses.

To help account for differences between tissues in the percent of all cells that are T cells, we normalized the total and intact HIV DNA levels to TCRα mRNA expression, as previously described [65, 66]. The total HIV DNA per TCRα mRNA was lower in PBMC than in ileum, colon or ectocervix (all FDR corrected *p* < 0.05; Figure 1C and Figure S3B). Moreover, the total HIV DNA/TCRα mRNA was greater in all gut tissues and ectocervix as compared to the endocervix or endometrium (all FDR corrected *p* < 0.05; Figure 1C and Figure S4B). In the limited number of women in whom we detected intact HIV DNA, there were no apparent differences between tissues in levels of intact HIV DNA/TCRα mRNA (Figure 1C).

Since CD8^+^ T cells will contribute to the total TCRα mRNA expression but are not usually infected by HIV, we further normalized the HIV DNA levels per TCRα mRNA by the percent of CD4^+^ T cells. With this normalization, the total HIV DNA tended to be higher in gut tissues compared to PBMC or endometrium (Figure S3B). These results suggest that the frequency of HIV‐infected CD4^+^ T cells is greater in the gut than in blood or endometrium.

### 3.3 HIV‐infected cells in the blood and endometrium transcribe more HIV than those in the gut, cervix and liver

To characterize the mechanisms that regulate HIV expression in the blood and tissues, we quantified read‐through, initiated (total), 5’elongated, polyadenylated and multiply spliced HIV transcripts in the PBMC and tissue samples (Figure 2A). To account for differences between tissues in the proportion of all cells that are HIV‐infected, we normalized the levels of each HIV transcript/million cells to the total HIV DNA/million cells (LTR target, corrected for 2 copies/provirus) to calculate the average level of each HIV transcript per provirus (or infected cell, assuming one provirus/cell) (Figure 2B). HIV transcription initiation (initiated HIV RNA per provirus) was higher in PBMC and endometrium than in ileum, colon, rectosigmoid, ectocervix or endocervix (all FDR‐corrected *p* < 0.05; Figure 2B and Figure S5). Initiated HIV transcripts per provirus were also higher in the endocervix than in the ileum, colon, rectum or ectocervix (all FDR‐corrected *p* < 0.05; Figure 2 and Figure S5), and higher in the rectum than in the ileum, colon or ectocervix (all FDR‐corrected *p* < 0.05; Figure 2 and Figure S5).

Likewise, levels of 5’elongated HIV RNA per provirus were higher in PBMC than in all gut sites, higher in endometrium than in ileum or colon, higher in the rectum than in ileum, and higher in the ileum than in endocervix (all FDR‐corrected *p* < 0.05; Figure 2 and Figure S5). However, when we measured the progression through 5’elongation (ratio of 5’elongated/initiated HIV RNA) using only the detectable values, 5’elongation tended to be greater in most tissues (except endometrium) than in PBMC (Figure 2C). Polyadenylated HIV transcripts were detected in PBMC from all six individuals and multiply spliced HIV transcripts were detected in PBMC from three of six individuals, whereas polyadenylated and multiply spliced HIV transcripts were rarely detected in the tissues (Figure 2B). The number of analysed cells was similar among all tissues except the endocervix (Figure S3C). These results suggest tissue‐specific differences in the mechanisms that govern HIV expression, with lower HIV transcription in most tissues (save endometrium) than blood.

**Figure 3:**
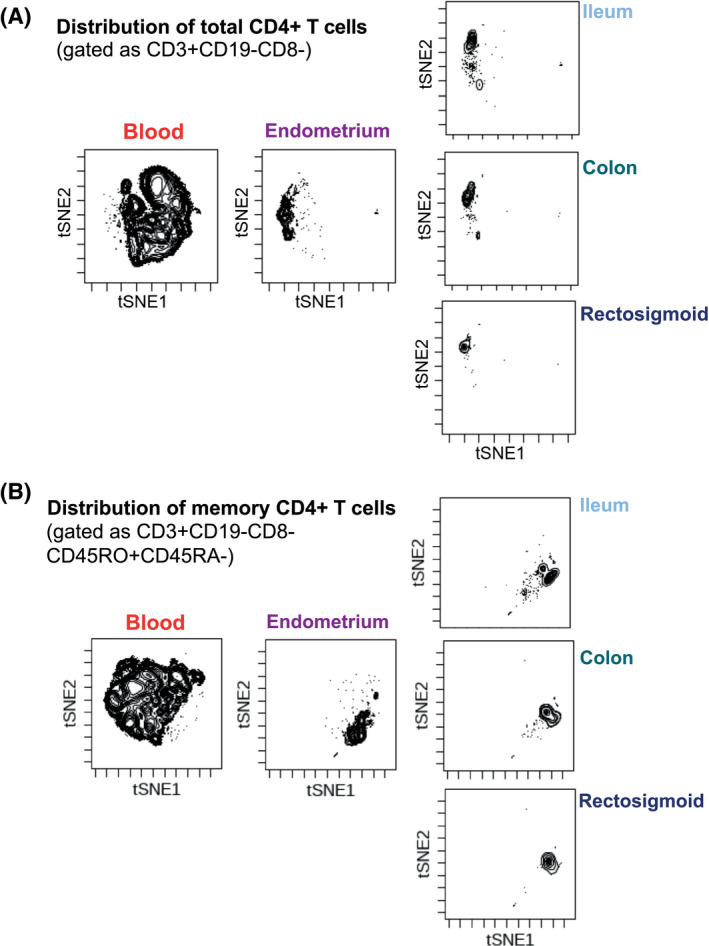
CD4^+^ T cells from the endometrium exhibit tissue‐ and not blood‐oriented signatures. Specimens from the blood, endometrium, ileum, colon and rectosigmoid of the same women were phenotyped using a 39‐parameter CyTOF panel, and then visualized by t‐SNE. Shown are results pre‐gated on **(A)** CD4^+^ T cells (identified as CD3^+^CD19^‐^CD8^−^ cells) or **(B)** memory CD4^+^ T cells (identified as CD3^+^CD19^‐^CD8^−^CD45RO^+^CD45RA^−^ cells). Data shown from one individual are representative of all participants. The endometrial CD4^+^ and memory CD4^+^ T cells reside in regions of the t‐SNE occupied by CD4^+^ T cells from gut, and distinct from the regions occupied by their blood counterparts, suggesting that few of the endometrial CD4^+^ T cells were from blood.

One explanation for why the transcriptional profile in the endometrium differed from the other tissues and was more similar to the PBMC is the possibility that the endometrium was heavily contaminated with blood‐derived cells, particularly since the endometrium is heavily vascularized. To test this possibility, we used CyTOF to compare the phenotype of immune cells from the endometrium to the phenotypes of immune cells from the blood and gut. The global phenotypes of the endometrial CD4^+^ T cells were different from those in the blood and more similar to those in the gut (Figure 3). These data suggest that the similarities in HIV transcription between PBMC and endometrium are not solely driven by blood in the endometrial biopsies. Interestingly, cellular proteins previously associated with HIV reservoir cells (PD1, CXCR5, CCR7, CD27, CCR6, CD69, CD103) [67, 68, 69, 70, 71] were detected within subsets of memory CD4+ T cells from all tissues, but expression levels seemed to vary between tissues (Figure S6).

## 4 DISCUSSION

We characterized the levels of total and intact HIV DNA, along with the levels of various HIV transcripts, in blood, gut, genital tract and liver from HIV‐positive ART‐treated women. To our knowledge, this is the first study to compare the HIV DNA levels (including both total and intact) and the HIV transcription profile among multiple tissues from ART‐treated women.

We detected total HIV DNA in all tissues and intact HIV DNA in blood, ileum, colon, rectosigmoid and ectocervix, demonstrating that the gut, genital tract and liver are all sites of HIV persistence in ART‐treated women. Furthermore, when normalized by T‐cell content, the total HIV DNA was higher in the gut and ectocervix than in the blood, endocervix, endometrium or liver. These data suggest that, as previously observed in HIV‐positive ART‐treated men [36, 72, 73], the frequency of HIV‐infected CD4^+^ T cells is higher in the gut than in the blood, and demonstrate for the first time that the frequency of HIV‐infected T cells may be higher in the ectocervix than endocervix or endometrium.

HIV transcription initiation was higher in blood and endometrium than in gut and cervical tissues. The differences between blood and gut resemble prior findings from HIV‐positive ART‐suppressed men [60], in whom HIV transcription initiation was higher in blood than in rectum. However, within the female gut, we observed that HIV transcription initiation was higher in the rectosigmoid than in the colon or ileum. These results differ from those observed in HIV‐positive ART‐suppressed men, in whom HIV transcription was higher in the ileum than in the right colon or rectum [32, 73]. Thus, our results may suggest differences between men and women in the mechanisms that govern HIV expression in different regions of the gut, although more specimens from additional donors will need to be analysed to confirm these findings. One possible explanation for this difference is that the rectum may be more likely to be the initial site of infection in HIV‐positive men who have sex with men, which could influence the characteristics of the infected cells that persist despite ART.

HIV transcription initiation was higher in endometrium than in other female genital tract tissues or gut, suggesting that there are regional differences within the female genital tract, with the endometrium being a “hotspot” for HIV transcription. Levels per provirus of elongated HIV transcripts were also higher in PBMC and endometrium than in gut and cervical tissues. The increased transcription in the endometrium did not appear to be the result of peripheral blood contamination. In contrast to initiated and elongated transcripts, polyadenylated and multiply spliced HIV transcripts were less frequently detected in all tissues, including the endometrium, compared to blood. If this finding does not just reflect sampling fewer total infected cells from the tissues, it may indicate greater constitutive blocks to HIV transcriptional completion and/or splicing in these tissues, which could further limit virus production in the tissues. Together, these data suggest that the blocks to HIV expression, and likely the mechanisms that govern HIV latency, differ between peripheral blood and tissues from the gut, genital tract and liver.

Limitations of the study should be acknowledged. First, the gut and genital tissues were available from only six participants, whereas liver biopsies were available only from three participants, which limited our ability to draw conclusions about the liver. Second, instead of purifying CD4^+^ T cells, whole tissue biopsies were used to measure HIV DNA and RNA, which would be expected to result in fewer infected cell equivalents per PCR well and less chance of detecting more rare HIV transcripts (such as multiply spliced HIV RNA). However, by avoiding tissue collagenase treatments and cell sorting prior to HIV DNA and RNA quantification, we were able to minimize the effect of sample processing on the HIV transcription profile. Moreover, by analysing whole tissue samples, we were also able to detect HIV DNA and RNA that may exist in cells other than T cells, such as myeloid cells [32, 35, 36, 74, 75, 76]. Third, the number of cells recovered per tissue sample differed depending on the type of tissue and number of biopsies collected. However, the input of cells analysed for HIV DNA and RNA was similar among all tissues except endocervix (Figure S3A and Figure S3C). Finally, while we detected statistically significant differences among tissues in HIV DNA and initiated (total) and 5’elongated HIV transcripts, it is difficult to exclude differences in intact proviruses or polyadenylated or multiply spliced transcripts due to the number of undetectable determinations.

## 5 CONCLUSIONS

Our findings demonstrate that blood, gut, liver and female genital tract are all sites of HIV persistence in ART‐treated women, and suggest tissue‐specific differences in the mechanisms that govern HIV latency, with less HIV transcription in most tissues than blood. These results have important implications for understanding HIV persistence and developing strategies aimed at HIV cure. Future studies should investigate the cellular and viral factors that regulate HIV latency in different tissue sanctuaries both in HIV‐positive men and women in order to find effective drugs aimed at disrupting HIV latency.

## Competing interest

The authors have declared no conflict of interest.

## Authors’ contributions

S.A.Y., N.R.R. and R.G. designed the study and obtained funding. J.P., N.E., R.G. and P.T. recruited participants and provided samples and clinical data. S.M.L. and G.X. performed experiments. P.K. assisted with sample processing. S.M.L., G.X., D.S., S.L., N.R.R. and S.A.Y. analysed data. S.A.Y., J.W. and N.R.R. supervised the work. S.M.L and S.A.Y. wrote the manuscript. All authors edited the manuscript. All authors have read and accepted the final manuscript.

## Supporting information


**Figure S1**. CONSORT study flow diagram.

**Figure S2**. Statistical analysis using Wilcoxon signed‐rank test. (A) Quantification of total (LTRs and 5’LTR) and intact (Ψ + Env) HIV DNA per million total tissue cells, (B) per TCRα mRNA expression (normalization by T cell content) and (C) levels of each HIV transcript per provirus (HIV RNA/DNA). Symbol colours denote different tissues; symbols/shapes show different participants; symbols without borders indicate values below the limit of detection; *p*‐values (Wilcoxon signed‐rank test) are represented in grey for *p* = 0.06 and black for *p* = 0.03.

**Figure S3**. Cells analysed per HIV DNA and RNA assay, and total and intact HIV reservoir size per TCRα and per percent of CD4 T cells. (A) Number of analysed cells per HIV DNA ddPCR reaction. (B) Quantification of total (LTRs and 5’LTR) and intact (Ψ + Env) HIV DNA per TCRα mRNA expression (normalization by T cell content) and per percent of all T cells that are CD4^+^ (measured by CyTOF in 5 out of the 6 participants). (C) Number of analysed cells per HIV RNA RT‐ddPCR reaction. Symbol colours denote different tissues; symbols/shapes show different participants; symbols without borders indicate values below the limit of quantification.

**Figure S4**. Multilevel mixed‐effects negative binomial regression *p*‐values of the comparison among tissues in HIV DNA. (A) Total HIV DNA (5’LTR and LTR) per cell. (B) Total HIV DNA per TCRα mRNA expression (PBMC: peripheral blood mononuclear cells; ILE: ileum; COL: colon; RS: rectosigmoid; CVX: ectocervix; ECC: endocervix; EMBx: endometrium). Significant *p* values are shown in yellow and green (*p ≤ 0.05, **p ≤ 0.001 and ***p ≤ 0.0001) and non‐significant *p* values are shown in blue (*p* > 0.05). Upper limit of colour scheme shows *p* > 0.10 (dark blue).

**Figure S5**. Multilevel mixed‐effects negative binomial regression *p*‐values of the comparison among tissues in HIV transcripts per provirus. RTh: read‐through HIV RNA; TAR: initiated HIV RNA; longLTR: 5’elongated HIV RNA; PolyA: polyadenylated HIV RNA; TatRev: multiply spliced HIV RNA; PBMC: peripheral blood mononuclear cells; ILE: ileum; COL: colon; RS: rectosigmoid; CVX: ectocervix; ECC: endocervix; EMBx: endometrium. Significant *p* values are shown in yellow and green (*p ≤ 0.05, **p ≤ 0.001 and ***p ≤ 0.0001) and non‐significant *p* values are shown in blue (*p* > 0.05). Upper limit of colour scheme shows *p* > 0.10.

**Figure S6**. tSNE depiction of CyTOF dataset of blood and tissues from representative donor showing expression profiles of antigens previously associated with HIV reservoir cells. (A) tSNE depiction of memory CD4+ T cells (CD3+CD19‐CD8‐CD45RO+CD45RA‐) isolated from blood or from biopsies from endometrium, ileum, colon or rectosigmoid, all procured during the same study visit. Expression levels of markers of T follicular helper (Tfh) cells (PD1, CXCR5), central memory (Tcm) cells (CCR7, CD27), Th17 cells (CCR6) and tissue‐resident memory (Trm) cells (CD69, CD103) are shown as heatmaps, with the highest expression depicted in red and lowest in blue. These subsets of memory CD4+ T cells have all previously shown to harbour HIV reservoir cells. Circled are some regions of the tSNE in the blood specimens that harbour high expression levels of these markers. The tSNE for all five specimens were run at the same time. (B) The tissue specimens in *panel A* were run in a separate tSNE without the blood specimen to better resolve the tissue cells. Note that all tissues expressed very high levels of at least one of the markers of reservoir cells.

**Table S1**. List of CyTOF antibodies used in this study
