# Supplementary figures and images for: Tissue‐specific differences in HIV DNA levels and mechanisms that govern HIV transcription in blood, gut, genital tract and liver in ART‐treated women
Source: J Int AIDS Soc. 2021 Jul 8;24(7):e25738. doi: 10.1002/jia2.25738 (PMC8264406; doi:10.1002/jia2.25738)

Study flow diagram

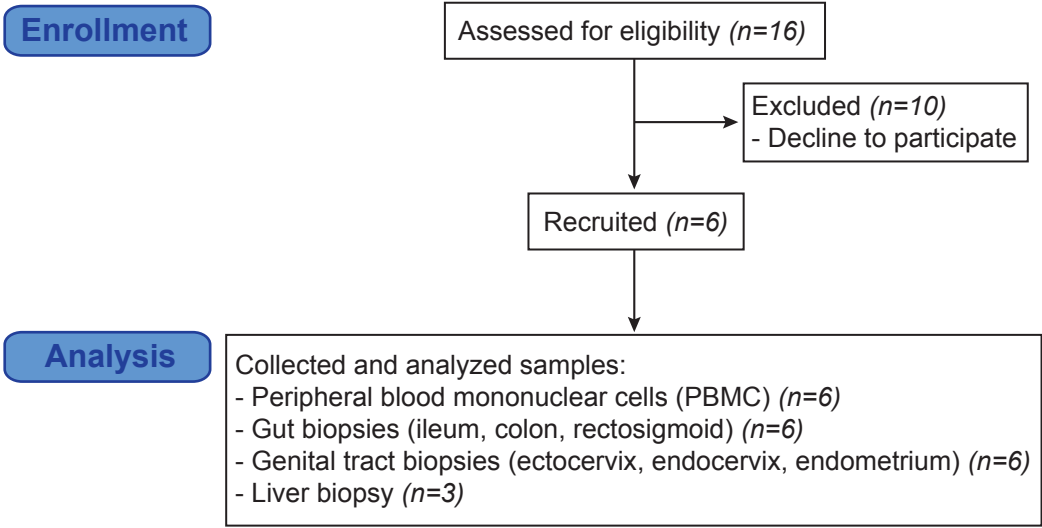

Supplement: Supplementary file 1 — Figure S1. CONSORT study flow diagram. [file JIA2-24-e25738-s002.pdf]

Supplementary Figure 2

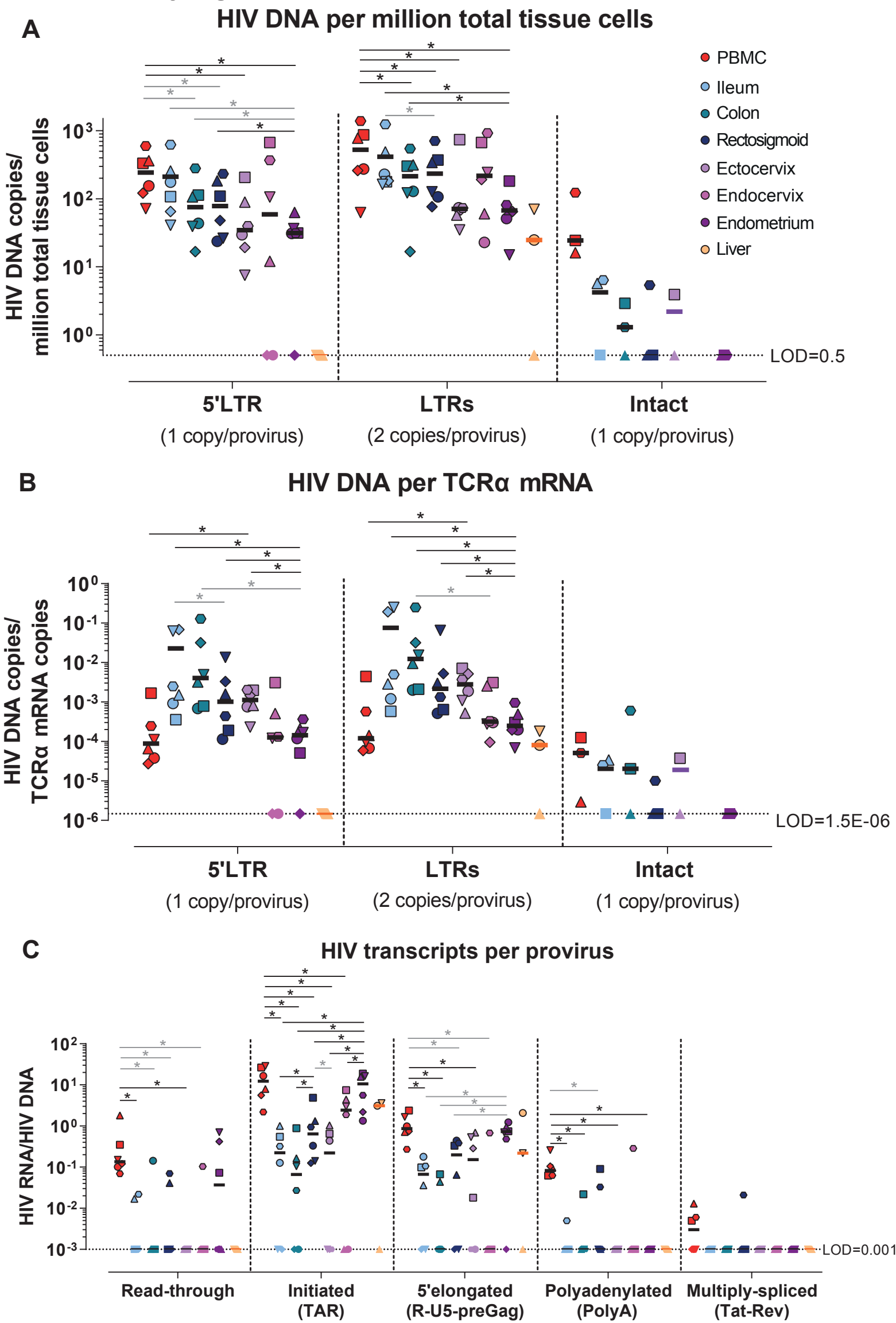

Supplement: Supplementary file 2 — Figure S2. Statistical analysis using Wilcoxon signed‐rank test. (A) Quantification of total (LTRs and 5’LTR) and intact (Ψ + Env) HIV DNA per million total tissue cells, (B) per TCRα mRNA expression (normalization by T cell content) and (C) levels of each HIV transcript per provirus (HIV RNA/DNA). Symbol colours denote different tissues; symbols/shapes show different participants; symbols without borders indicate values below the limit of detection; p‐values (Wilcoxon signed‐rank test) are represented in grey for p = 0.06 and black for p = 0.03. [file JIA2-24-e25738-s007.pdf]

Supplementary Figure 3

A

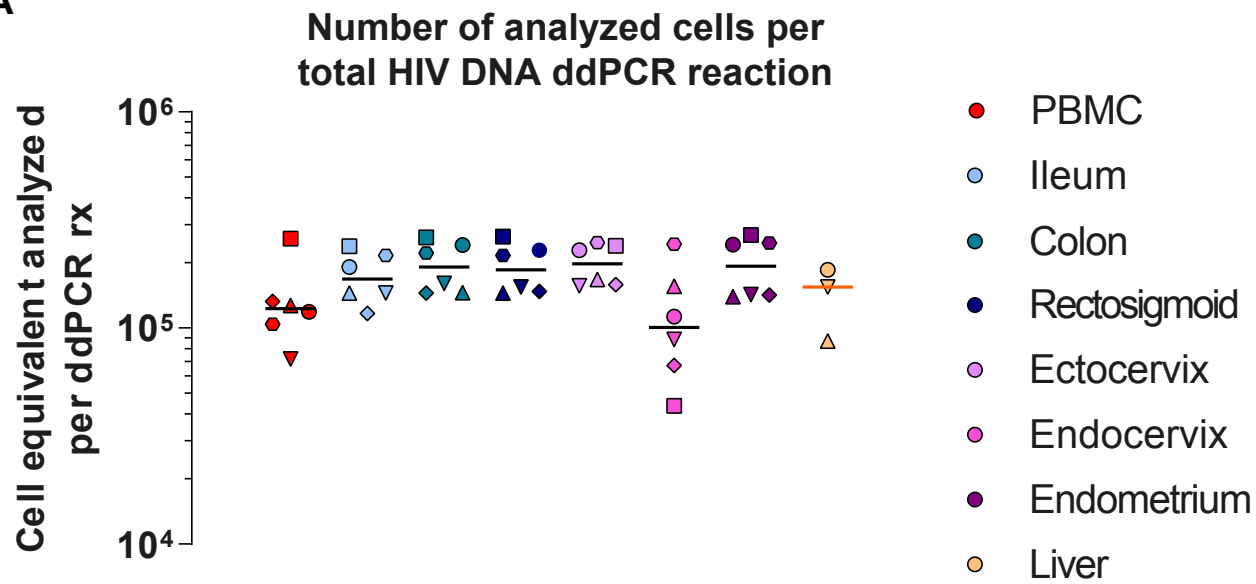

B

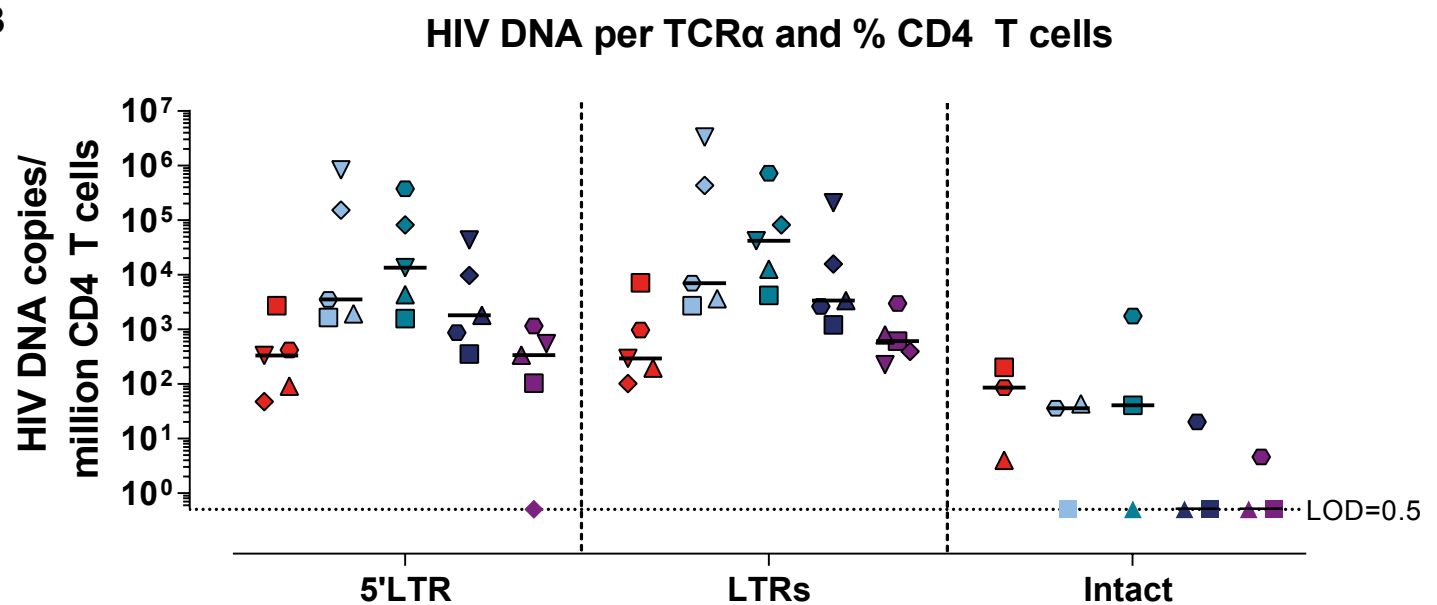

C

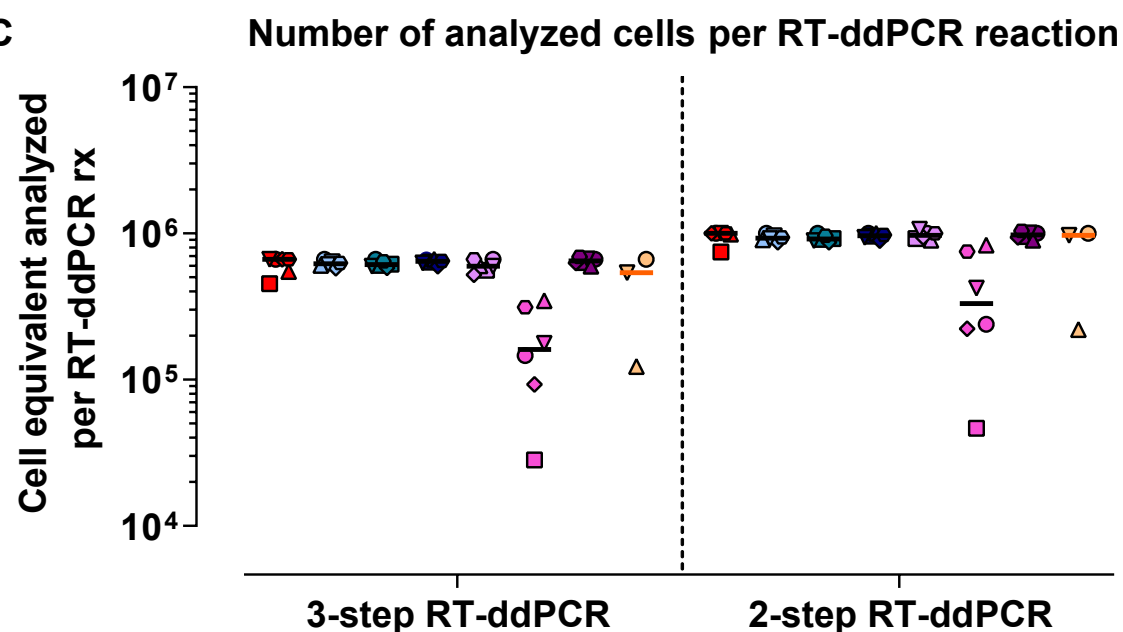

Supplement: Supplementary file 3 — Figure S3. Cells analysed per HIV DNA and RNA assay, and total and intact HIV reservoir size per TCRα and per percent of CD4 T cells. (A) Number of analysed cells per HIV DNA ddPCR reaction. (B) Quantification of total (LTRs and 5’LTR) and intact (Ψ + Env) HIV DNA per TCRα mRNA expression (normalization by T cell content) and per percent of all T cells that are CD4+ (measured by CyTOF in 5 out of the 6 participants). (C) Number of analysed cells per HIV RNA RT‐ddPCR reaction. Symbol colours denote different tissues; symbols/shapes show different participants; symbols without borders indicate values below the limit of quantification. [file JIA2-24-e25738-s003.pdf]

Supplementary Figure 6

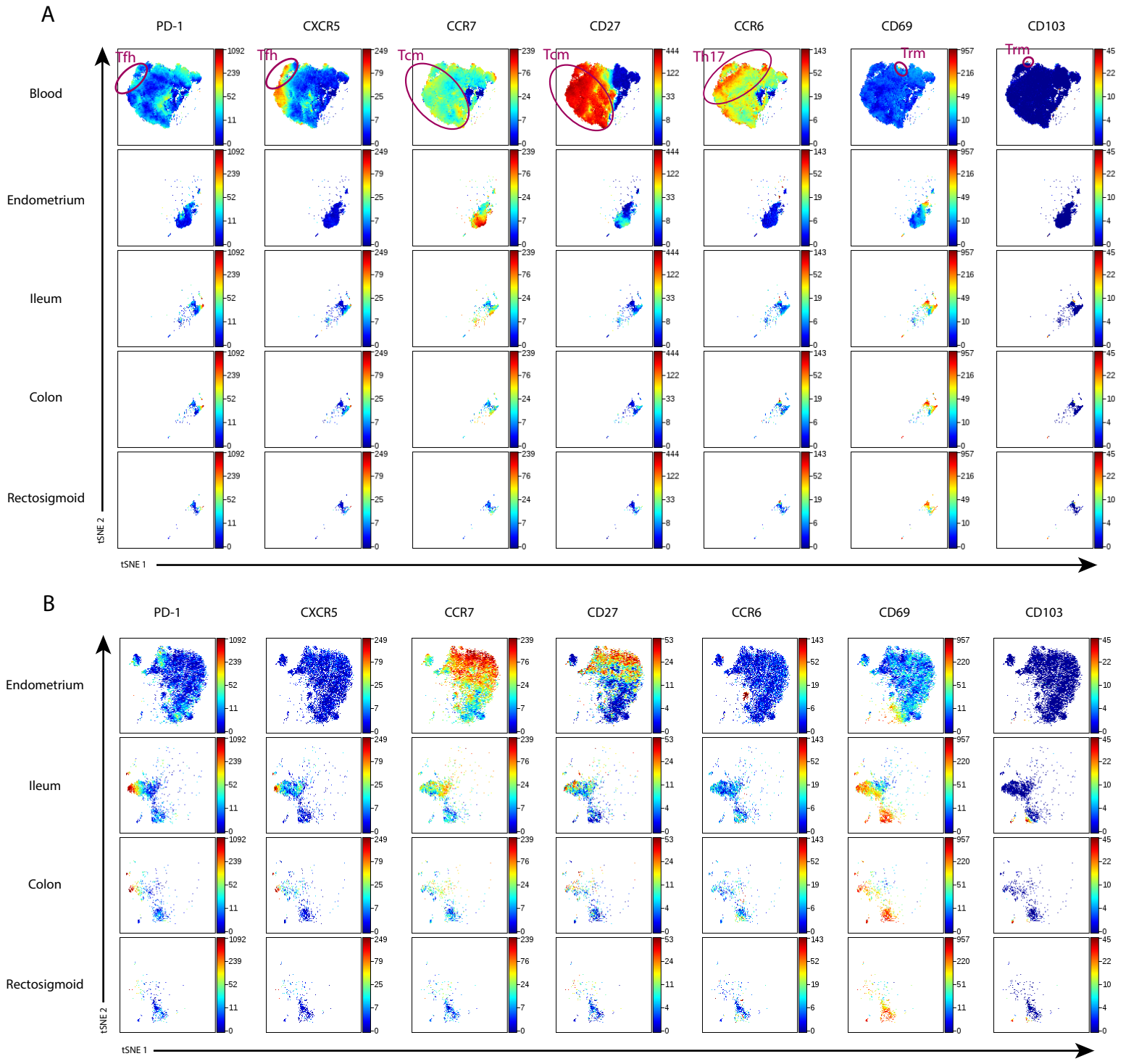

Supplement: Supplementary file 6 — Figure S6. tSNE depiction of CyTOF dataset of blood and tissues from representative donor showing expression profiles of antigens previously associated with HIV reservoir cells. (A) tSNE depiction of memory CD4+ T cells (CD3+CD19‐CD8‐CD45RO+CD45RA‐) isolated from blood or from biopsies from endometrium, ileum, colon or rectosigmoid, all procured during the same study visit. Expression levels of markers of T follicular helper (Tfh) cells (PD1, CXCR5), central memory (Tcm) cells (CCR7, CD27), Th17 cells (CCR6) and tissue‐resident memory (Trm) cells (CD69, CD103) are shown as heatmaps, with the highest expression depicted in red and lowest in blue. These subsets of memory CD4+ T cells have all previously shown to harbour HIV reservoir cells. Circled are some regions of the tSNE in the blood specimens that harbour high expression levels of these markers. The tSNE for all five specimens were run at the same time. (B) The tissue specimens in panel A were run in a separate tSNE without the blood specimen to better resolve the tissue cells. Note that all tissues expressed very high levels of at least one of the markers of reservoir cells. [file JIA2-24-e25738-s004.pdf]
