# Supplementary material for: Tissue‐specific differences in HIV DNA levels and mechanisms that govern HIV transcription in blood, gut, genital tract and liver in ART‐treated women
Source: J Int AIDS Soc. 2021 Jul 8;24(7):e25738. doi: 10.1002/jia2.25738 (PMC8264406; doi:10.1002/jia2.25738)

Supplementary Figure 4

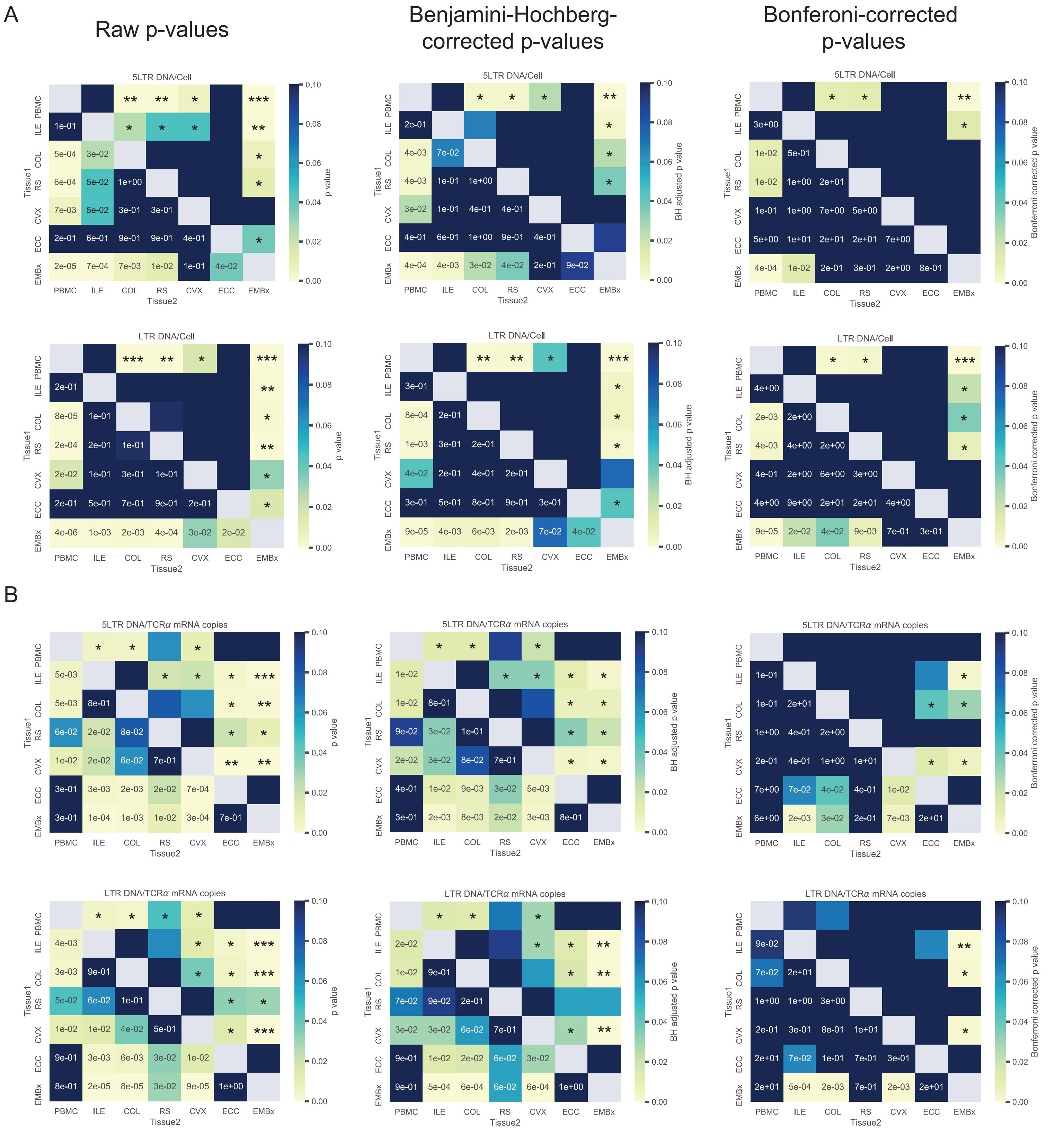

Supplement: Supplementary file 4 — Figure S4. Multilevel mixed‐effects negative binomial regression p‐values of the comparison among tissues in HIV DNA. (A) Total HIV DNA (5’LTR and LTR) per cell. (B) Total HIV DNA per TCRα mRNA expression (PBMC: peripheral blood mononuclear cells; ILE: ileum; COL: colon; RS: rectosigmoid; CVX: ectocervix; ECC: endocervix; EMBx: endometrium). Significant p values are shown in yellow and green (*p ≤ 0.05, **p ≤ 0.001 and ***p ≤ 0.0001) and non‐significant p values are shown in blue (p > 0.05). Upper limit of colour scheme shows p > 0.10 (dark blue). [file JIA2-24-e25738-s001.pdf]
