# Supplementary material for: Tissue‐specific differences in HIV DNA levels and mechanisms that govern HIV transcription in blood, gut, genital tract and liver in ART‐treated women
Source: J Int AIDS Soc. 2021 Jul 8;24(7):e25738. doi: 10.1002/jia2.25738 (PMC8264406; doi:10.1002/jia2.25738)

Supplementary Figure 5

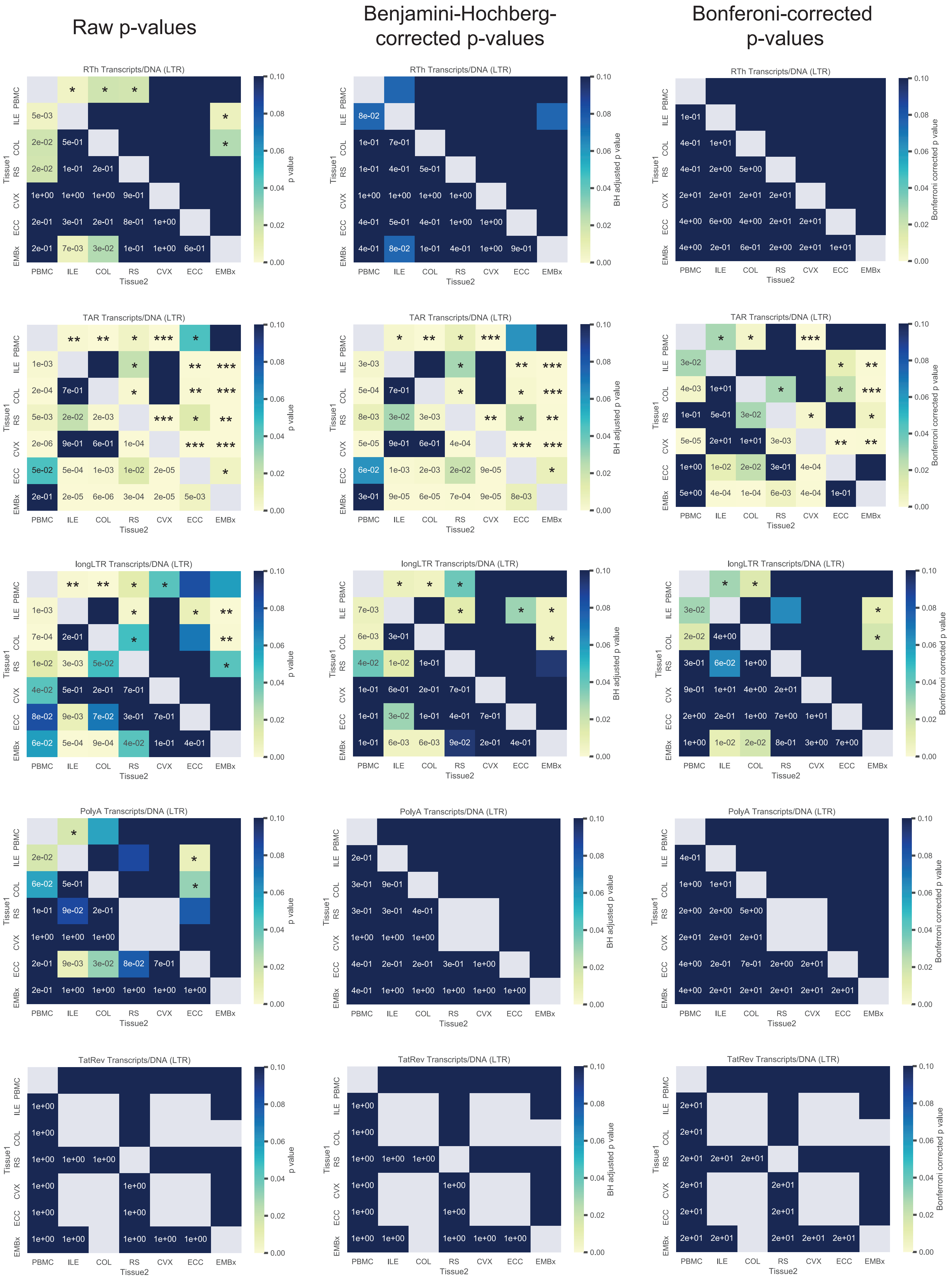

Supplement: Supplementary file 5 — Figure S5. Multilevel mixed‐effects negative binomial regression p‐values of the comparison among tissues in HIV transcripts per provirus. RTh: read‐through HIV RNA; TAR: initiated HIV RNA; longLTR: 5’elongated HIV RNA; PolyA: polyadenylated HIV RNA; TatRev: multiply spliced HIV RNA; PBMC: peripheral blood mononuclear cells; ILE: ileum; COL: colon; RS: rectosigmoid; CVX: ectocervix; ECC: endocervix; EMBx: endometrium. Significant p values are shown in yellow and green (*p ≤ 0.05, **p ≤ 0.001 and ***p ≤ 0.0001) and non‐significant p values are shown in blue (p > 0.05). Upper limit of colour scheme shows p > 0.10. [file JIA2-24-e25738-s006.pdf]
