# Supplementary material for: Tissue‐specific differences in HIV DNA levels and mechanisms that govern HIV transcription in blood, gut, genital tract and liver in ART‐treated women
Source: J Int AIDS Soc. 2021 Jul 8;24(7):e25738. doi: 10.1002/jia2.25738 (PMC8264406; doi:10.1002/jia2.25738)
Supplement: Supplementary file 7 — Table S1. List of CyTOF antibodies used in this study [file JIA2-24-e25738-s005.docx]

| **Supplementary Table 1.** List of CyTOF antibodies used in this study. Antibodies were either purchased from the indicated vendor or prepared in-house using commercially available MaxPAR conjugation kits per manufacturer’s instructions (Fluidigm). | | | |
| --- | --- | --- | --- |
| **Antigen Target** | **Clone** | **Elemental Isotope** | **Vendor** |
| HLADR | TU36 | Qdot (112Cd) | Life Technologies |
| RORγt* | AFKJS-9 | 115In | In-house |
| CD49d(α4) | 9F10 | 141Pr | Fluidigm |
| CD19 | HIB19 | 142Nd | Fluidigm |
| CD57 | HNK-1 | 143Nd | In-house |
| CCR5 | NP6G4 | 144Nd | Fluidigm |
| CTLA-4* | 14D3 | 145Nd | In-house |
| CD8 | RPA-T8 | 146Nd | Fluidigm |
| CD7 | CD76B7 | 147Sm | Fluidigm |
| ICOS | C398.4A | 148Nd | Fluidigm |
| Tbet* | 4B10 | 149Sm | In-house |
| Gag(KC57)* | FH190-1-1 | 150Nd | In-house |
| CD103 | Ber-ACT8 | 151Eu | Fluidigm |
| TCRγδ | 11F2 | 152Sm | Fluidigm |
| CD62L | DREG56 | 153Eu | Fluidigm |
| TIGIT | MBSA43 | 154Sm | Fluidigm |
| CCR6 | 11A9 | 155Gd | In-house |
| CD29(β1) | TS2/16 | 156Gd | Fluidigm |
| OX40 | ACT35 | 158Gd | Fluidigm |
| CCR7 | G043H7 | 159Tb | Fluidigm |
| CD28 | CD28.2 | 160Gd | Fluidigm |
| CD45RO | UCHL1 | 161Dy | In-house |
| CD69 | FN50 | 162Dy | Fluidigm |
| CRTH2 | BM16 | 163Dy | Fluidigm |
| PD-1 | EH12.1 | 164Dy | In-house |
| CD127 | A019D5 | 165Ho | Fluidigm |
| CXCR5 | RF8B2 | 166Er | In-house |
| CD27 | L128 | 167Er | Fluidigm |
| CD30 | BERH8 | 168Er | In-house |
| CD45RA | HI100 | 169Tm | Fluidigm |
| CD3 | UCHT1 | 170Er | Fluidigm |
| Gag (mixture)* | 71-31, 91-5, 241-D, AG3.0 | 171Yb | In-house |
| CD38 | HIT2 | 172Yb | Fluidigm |
| α4β7 | Act1 | 173Yb | In-house |
| CD4 | SK3 | 174Yb | Fluidigm |
| CXCR4 | 12G5 | 175Lu | Fluidigm |
| CD25 | M-A251 | 176Yb | In-house |
| Live/Dead |  | 195Pt | Sigma-Aldrich |
| DNA |  | 191Ir & 193Ir | Fluidigm |
| *: Intracellular antibodies | |  |  |
